# Crystal structure of tetra­kis­(μ-2,4,6-tri­methyl­benzoato-κ^2^ *O*:*O*′)bis­[(nicotinamide-κ*N* ^1^)copper(II)]

**DOI:** 10.1107/S2056989015013882

**Published:** 2015-07-31

**Authors:** Gülçin Şefiye Aşkın, Hacali Necefoğlu, Safiye Özkaya, Nefise Dilek, Tuncer Hökelek

**Affiliations:** a Department of Physics, Hacettepe University, 06800 Beytepe, Ankara, Turkey; b Department of Chemistry, Kafkas University, 36100 Kars, Turkey; c International Scientific Research Centre, Baku State University, 1148 Baku, Azerbaijan; d Aksaray University, Department of Physics, 68100, Aksaray, Turkey

**Keywords:** crystal structure, copper(II) complex, nicotinamide ligand, 2,4,6-tri­methyl­benzoate

## Abstract

In the title binuclear Cu^II^ complex, [Cu_2_(C_10_H_11_O_2_)_4_(C_6_H_6_N_2_O)_2_], the two Cu^II^ cations [Cu⋯Cu = 2.5990 (5) Å] are bridged by four 2,4,6-tri­methyl­benzoate (TMB) anions. The four nearest O atoms around each Cu^II^ cation form distorted square-planar arrangements and the distorted square-pyramidal coordinations are completed by the pyridine N atoms of nicotinamide mol­ecules at distances of 2.164 (2) and 2.165 (2) Å, respectively. The Cu^II^ cations are displaced by −0.2045 (3) and 0.2029 (3) Å from the corresponding planes formed by the nearest four O atoms. In the mol­ecule, the dihedral angles between the planes of the benzene rings and the adjacent carboxyl­ate groups are 80.6 (2), 51.4 (2), 24.4 (2) and 32.5 (2)°, while the planes of the pyridine rings are oriented at a dihedral angle of 11.28 (10)°. In the crystal, bifurcated N—H⋯O and weak C—H⋯O hydrogen bonds link the mol­ecules, enclosing *R*_2_^2^(8) and *R*_4_^4^(8) ring motifs, into a three-dimensional network. The structure contains a solvent-accessible void of 72 Å^3^, but there is no solvent mol­ecule located within this void. The crystal studied was an inversion twin refined with a minor component of 0.488 (8).

## Abstract 2

Four carboxyl­ate groups of 2,4,6-tri­methyl­benzoate anions bridge two Cu^II^ cations to form a binuclear complex. Distorted square-pyramidal coordinations are completed by the pyridine N atoms of nicotinamide mol­ecules.

## Chemical context   

Nicotinamide (NA) is one form of niacin. A deficiency of this vitamin leads to loss of copper from the body, known as pellagra disease. Victims of pellagra show unusually high serum and urinary copper levels (Krishnamachari, 1974 ▸). It is thus of inter­est to determine the manner in which copper inter­acts with niacin and nicotinamide. In the structures of some complexes obtained from the reactions of Cu^II^ ions with NA, *e.g.* [Cu(sal)_2_(NA)_2_] (sal is salicylate) (Hoang *et al.*, 1993 ▸) and [Cu(C_7_H_3_ClFO_2_)_2_(NA)_2_] (Hoang *et al.*, 1995 ▸), NA is a monodentate ligand coordinating to Cu^II^ *via* its pyridine N atom. In its rare earth complexes, NA coordinates to the rare earth ion *via* only the O atoms of the substituents, not by the pyridine N atom (Poray-Koshits *et al.*, 1976 ▸). Coordination *via* the amide N atom may also occur. Hence, NA may form mol­ecular or polymeric structures affecting such properties of the compounds as their solubility.

The structure–function–coordination relationships of the aryl­carboxyl­ate ion in Cu^II^ complexes of benzoic acid derivatives may change depending on the nature and position of the substituted groups on the benzene ring, the nature of the additional ligand mol­ecule or solvent, and the pH and temperature of synthesis as in Zn^II^ complexes of benzoic acid derivatives (Shnulin *et al.*, 1981 ▸; Nadzhafov *et al.*, 1981 ▸; Antsyshkina *et al.*, 1980 ▸; Adiwidjaja *et al.*, 1978 ▸). When pyridine and its derivatives are used instead of water mol­ecules, the structure is completely different (Catterick *et al.*, 1974 ▸). In this context, we synthesized a Cu^II^-containing compound with 2,4,6-tri­methyl­benzoate (TMB) and NA ligands, namely tetra­kis­(μ-2,4,6-tri­methyl­benzoato-κ^2^ *O*:*O*′)bis­[(nicotinamide-κ*N* ^1^)copper(II)], [Cu_2_(TMB)_4_(NA)_2_], and report herein its crystal structure.
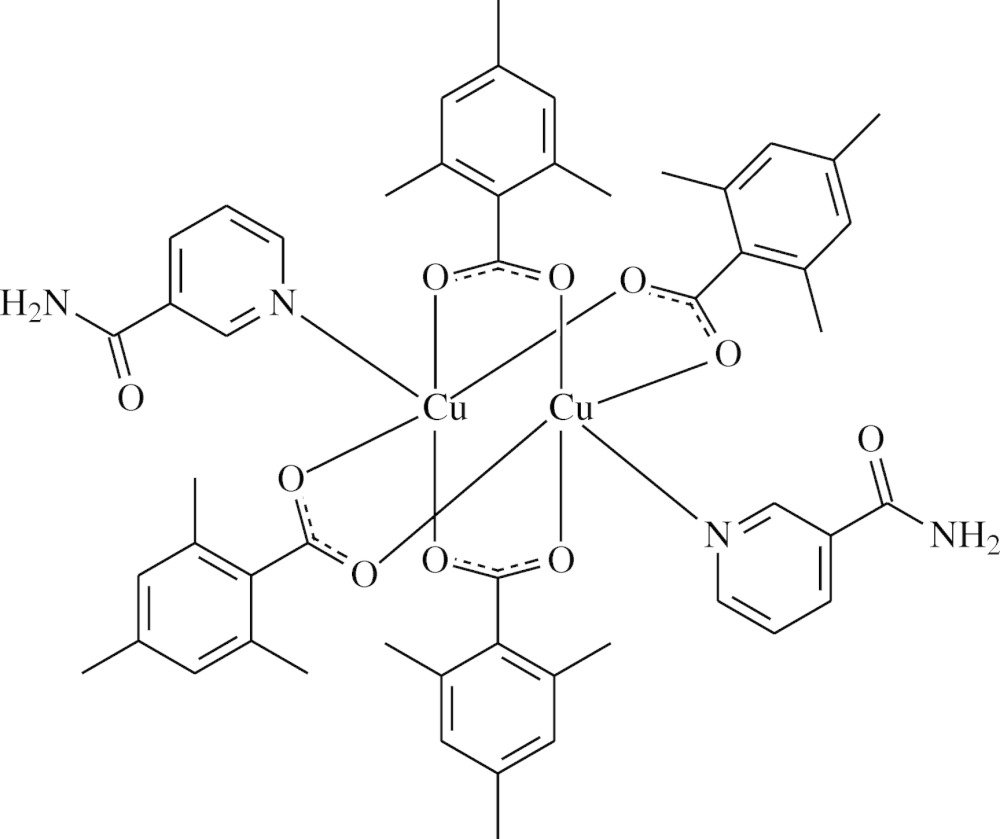


## Structural commentary   

**Figure 1:**
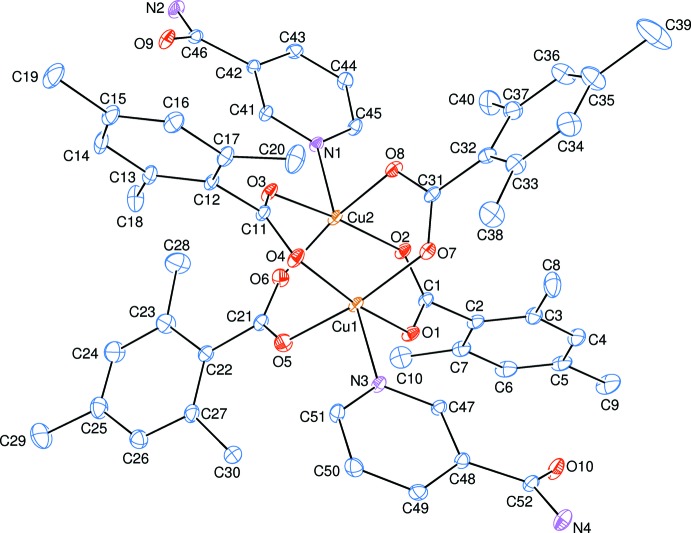
The mol­ecular structure of the title complex, showing the atom-numbering scheme. Displacement ellipsoids are drawn at the 50% probability level. H atoms have been omitted for clarity.

The binuclear title complex, [Cu_2_(TMB)_4_(NA)_2_], contains two Cu^II^ atoms surrounded by four TMB and two NA ligands (Fig. 1 ▸). The TMB groups act as bidentate bridging ligands. The Cu1⋯Cu2 [2.5990 (5) Å] distance is shorter than in [Cu_2_(C_6_H_5_COO)_4_(C_10_H_14_N_2_O)_2_] [2.613 (1) Å; Hökelek *et al.*, 1995 ▸], [Cu_2_(C_8_H_7_O_2_)_4_(C_6_H_6_N_2_O)_2_] [2.6375 (6)Å; Necefoğlu *et al.*, 2010 ▸], [Cu_2_(C_6_H_5_COO)_4_(py)_2_] [py is pyridine; 2.681 (1) Å; Usubaliev *et al.*, 1980 ▸] and [Cu_2_(CH_3_COO)_4_(H_2_O)_2_] (2.64 Å; van Niekerk & Schoening, 1953 ▸). In metallic copper, the Cu—Cu bond length is 2.55 Å (Lee, 1986 ▸). The title complex has the smallest Cu⋯Cu distance after metallic copper. Therefore, a weak orbital inter­action may exist between the two Cu atoms.

The average Cu—O distance is 1.972 (10) Å (Table 1 ▸) and four O atoms (O1/O4/O5/O7 and O2/O3/O6/O7) of the bridging TMB ligands around each Cu atom (Cu1 and Cu2) form distorted square-planar arrangements. The Cu1 and Cu2 atoms lie 0.2045 (3) Å below and 0.2029 (3) Å above the corresponding least-squares planes formed by the nearest O atoms, respectively. The average O—Cu—O bond angles are the same (89.4°) for both of Cu atoms. The distorted square-pyramidal coordination around each Cu atom (Cu1 and Cu2) is completed by the N atoms (N3 and N1) of the NA ligands (Table 1 ▸). The N3—Cu1⋯Cu2 and N1—Cu2⋯Cu1 angles are 176.46 (6) and 174.66 (7)°, respectively, and the dihedral angle between plane through atoms Cu1, O1, O2, C1, Cu2, O3, O4 and C11, and that through atoms Cu1, O5, O6, C21, Cu2, O7, O8 and C31 is 87.88 (3)°.

The near equalities of the C—O bonds in the carboxyl­ate groups (Table 1 ▸) indicate delocalized bonding arrangements, rather than localized single and double bonds. Bond lengths and angles are in good agreement with the values reported for other copper complexes: [Cu(CH_3_CO_2_)_2_(py)]_2_ (Barclay & Kennard, 1961 ▸; Hanic *et al.*, 1964 ▸), [Cu(CH_2_ClCO_2_)_2_(2Me-py)]_2_ (2Me-py is 2-methyl­pyridine; Davey & Stephens, 1970 ▸), [Cu_2_(CH_3_CO_2_)_4_(pyrazine)] (Morosin *et al.*, 1975 ▸) and [Cu(C_6_H_5_CO_2_)_2_(py)]_2_ (Speier & Fulop, 1989 ▸).

The dihedral angles between planar carboxyl­ate groups O1/O2/C1, O3/O4/C11, O5/O6/C21 and O7/O8/C31 and the adjacent benzene rings *A* (C2–C7), *B* (C12–C17), *C* (C22–C27) and *D* (C32–C37) are 80.6 (2), 51.4 (2), 24.4 (2) and 32.5 (2)°, respectively, while those between rings *A*, *B*, *C*, *D*, *E* (N1/C41–C45) and *F* (N3/C47–C51) are *A*/*B* = 11.68 (12), *A*/*C* = 83.97 (12), *A*/*D* = 69.30 (11), *A*/*E* = 79.41 (11), *A*/*F* = 74.72 (10), *B*/*C* = 84.41 (12), *B*/*D* = 73.91 (13), *B*/*E* = 70.46 (11), *B*/*F* = 67.39 (10), *C*/*D* = 34.92 (13), *C*/*E* = 51.82 (11), *C*/*F* = 43.92 (12), *D*/*E* = 69.74 (11), *D*/*F* = 58.56 (10) and *E*/*F* = 11.28 (10)°.

## Supra­molecular features   

**Figure 2:**
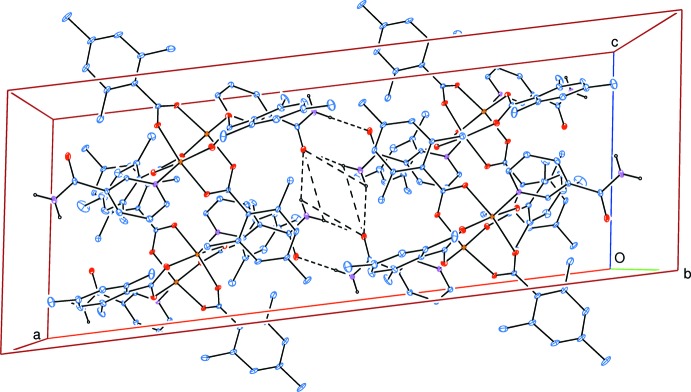
Part of the crystal structure viewed down [010]. Only inter­molecular N—H⋯O hydrogen bonds are shown as dashed lines, enclosing 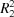
(8) and 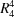
(8) ring motifs. Nonbonding H atoms have been omitted for clarity.

In the crystal, bifurcated N—H⋯O_n_ (n = nicotinamide) and C—H_py_⋯O_c_ (py = pyridine and c = carboxyl­ate) hydrogen bonds (Table 2 ▸) link the mol­ecules, enclosing 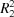
(8) and 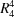
(8) ring motifs (Bernstein *et al.*, 1995 ▸) into a three-dimensional network (Fig. 2 ▸).

## Refinement   

The experimental details including the crystal data, data collection and refinement are summarized in Table 3 ▸. N- and C-bound H atoms were positioned geometrically, with N—H = 0.86 Å (for NH_2_) and C—H = 0.93 and 0.96 Å for aromatic and methyl H atoms, respectively, and constrained to ride on their parent atoms, with *U*_iso_(H) = *kU*_eq_(C,N), where *k* = 1.5 for methyl H atoms and *k* = 1.2 for NH_2_ and aromatic H atoms.

## Synthesis and crystallization   

The title compound was prepared by the reaction of CuSO_4_ (0.40 g, 2.5 mmol) in H_2_O (100 ml) and nicotinamide (0.61 g, 5 mmol) in H_2_O (25 ml) with sodium 2,4,6-tri­methyl­benzoate (0.93 g, 5 mmol) in H_2_O (150 ml). The mixture was set aside to crystallize at ambient temperature for three weeks, giving green single crystals.

## Supplementary Material

Crystal structure: contains datablock(s) I, global. DOI: 10.1107/S2056989015013882/xu5858sup1.cif

Structure factors: contains datablock(s) I. DOI: 10.1107/S2056989015013882/xu5858Isup2.hkl

CCDC reference: 1414247

Additional supporting information:  crystallographic information; 3D view; checkCIF report

## Figures and Tables

**Table 1:** Selected bond lengths ()

Cu1O1	1.9874(18)	Cu2N1	2.165(2)
Cu1O4	1.970(2)	O1C1	1.258(4)
Cu1O5	1.958(2)	O2C1	1.253(4)
Cu1O7	1.967(2)	O3C11	1.254(3)
Cu1N3	2.164(2)	O4C11	1.258(3)
Cu2O2	1.9611(18)	O5C21	1.262(4)
Cu2O3	1.9671(18)	O6C21	1.248(4)
Cu2O6	1.983(3)	O7C31	1.258(4)
Cu2O8	1.981(2)	O8C31	1.268(4)

**Table 2:** Hydrogen-bond geometry (, )

*D*H*A*	*D*H	H*A*	*D* *A*	*D*H*A*
N2H2*A*O10^i^	0.86	2.06	2.906(4)	170
N4H4*A*O9^ii^	0.86	2.10	2.955(4)	173
N4H4*B*O9^iii^	0.86	2.44	3.228(4)	153
C50H50O8^iii^	0.93	2.54	3.448(4)	166

Symmetry codes: (i) 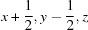; (ii) 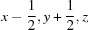; (iii) 
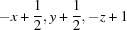
.

**Table 3:** Experimental details

Crystal data	
Chemical formula	[Cu_2_(C_10_H_11_O_2_)_4_(C_6_H_6_N_2_O)_2_]
*M*_r_	1024.11
Crystal system, space group	Monoclinic, *C*2
Temperature (K)	296
*a*, *b*, *c* ()	27.9186(7), 17.2843(5), 10.7570(3)
()	98.204(2)
*V* (^3^)	5137.7(2)
*Z*	4
Radiation type	Mo *K*
(mm^1^)	0.89
Crystal size (mm)	0.45 0.38 0.23

Data collection	
Diffractometer	Bruker SMART BREEZE CCD
Absorption correction	Multi-scan (*SADABS*; Bruker, 2012 ▸)
*T*_min_, *T*_max_	0.62, 0.91
No. of measured, independent and observed [*I* > 2(*I*)] reflections	64770, 12762, 9682
*R*_int_	0.055
(sin /)_max_ (^1^)	0.672

Refinement	
*R*[*F* ^2^ > 2(*F* ^2^)], *wR*(*F* ^2^), *S*	0.042, 0.099, 1.03
No. of reflections	12762
No. of parameters	626
No. of restraints	1
H-atom treatment	H-atom parameters constrained
_max_, _min_ (e ^3^)	0.65, 0.27
Absolute structure	Flack (1983 ▸), 5232 Friedel pairs
Absolute structure parameter	0.488(8)

Computer programs: *APEX2* and *SAINT* (Bruker, 2012 ▸), *SHELXS97* and *SHELXL97* (Sheldrick, 2008 ▸), *ORTEP-3 for Windows* and *WinGX* (Farrugia, 2012 ▸) and *PLATON* (Spek, 2009 ▸).

## supplementary crystallographic information

### Crystal data

**Table:**

[Cu_2_(C_10_H_11_O_2_)_4_(C_6_H_6_N_2_O)_2_]	*F*(000) = 2136
*M**_r_* = 1024.11	*D*_x_ = 1.324 Mg m^−^^3^
Monoclinic, *C*2	Mo *K*α radiation, λ = 0.71073 Å
Hall symbol: C 2y	Cell parameters from 9983 reflections
*a* = 27.9186 (7) Å	θ = 2.2–28.1°
*b* = 17.2843 (5) Å	µ = 0.89 mm^−^^1^
*c* = 10.7570 (3) Å	*T* = 296 K
β = 98.204 (2)°	Block, green
*V* = 5137.7 (2) Å^3^	0.45 × 0.38 × 0.23 mm
*Z* = 4	

### Data collection

**Table:**

Bruker SMART BREEZE CCD diffractometer	12762 independent reflections
Radiation source: fine-focus sealed tube	9682 reflections with *I* > 2σ(*I*)
Graphite monochromator	*R*_int_ = 0.055
φ and ω scans	θ_max_ = 28.5°, θ_min_ = 1.4°
Absorption correction: multi-scan (*SADABS*; Bruker, 2012)	*h* = −37→37
*T*_min_ = 0.62, *T*_max_ = 0.91	*k* = −23→23
64770 measured reflections	*l* = −14→14

### Refinement

**Table:**

Refinement on *F*^2^	Secondary atom site location: difference Fourier map
Least-squares matrix: full	Hydrogen site location: inferred from neighbouring sites
*R*[*F*^2^ > 2σ(*F*^2^)] = 0.042	H-atom parameters constrained
*wR*(*F*^2^) = 0.099	*w* = 1/[σ^2^(*F*_o_^2^) + (0.0515*P*)^2^ + 0.2754*P*] where *P* = (*F*_o_^2^ + 2*F*_c_^2^)/3
*S* = 1.02	(Δ/σ)_max_ = 0.001
12762 reflections	Δρ_max_ = 0.65 e Å^−^^3^
626 parameters	Δρ_min_ = −0.27 e Å^−^^3^
1 restraint	Absolute structure: Flack (1983), 5232 Friedel pairs
Primary atom site location: structure-invariant direct methods	Absolute structure parameter: 0.488 (8)

### Special details

**Table:**

Geometry. All esds (except the esd in the dihedral angle between two l.s. planes) are estimated using the full covariance matrix. The cell esds are taken into account individually in the estimation of esds in distances, angles and torsion angles; correlations between esds in cell parameters are only used when they are defined by crystal symmetry. An approximate (isotropic) treatment of cell esds is used for estimating esds involving l.s. planes.
Refinement. Refinement of F^2^ against ALL reflections. The weighted R-factor wR and goodness of fit S are based on F^2^, conventional R-factors R are based on F, with F set to zero for negative F^2^. The threshold expression of F^2^ > 2sigma(F^2^) is used only for calculating R-factors(gt) etc. and is not relevant to the choice of reflections for refinement. R-factors based on F^2^ are statistically about twice as large as those based on F, and R- factors based on ALL data will be even larger.

### Fractional atomic coordinates and isotropic or equivalent isotropic displacement parameters (Å^2^)

**Table:**

	*x*	*y*	*z*	*U*_iso_*/*U*_eq_	
Cu1	0.239902 (12)	0.411564 (19)	0.30231 (3)	0.03970 (9)	
Cu2	0.280625 (12)	0.30456 (2)	0.18371 (3)	0.04180 (10)	
O1	0.20128 (7)	0.42725 (13)	0.13420 (18)	0.0509 (6)	
O2	0.22783 (7)	0.32167 (13)	0.04546 (18)	0.0515 (6)	
O3	0.32599 (7)	0.29941 (16)	0.34120 (17)	0.0535 (5)	
O4	0.28428 (8)	0.37737 (14)	0.45109 (19)	0.0527 (6)	
O5	0.29283 (9)	0.47641 (15)	0.2605 (2)	0.0615 (6)	
O6	0.31786 (9)	0.39256 (15)	0.1280 (2)	0.0596 (7)	
O7	0.19297 (7)	0.33013 (14)	0.3270 (2)	0.0507 (6)	
O8	0.23743 (8)	0.23502 (14)	0.2633 (2)	0.0541 (6)	
O9	0.44557 (8)	0.12811 (17)	0.2878 (2)	0.0652 (6)	
O10	0.06281 (8)	0.55162 (17)	0.2345 (3)	0.0716 (7)	
N1	0.31634 (9)	0.21015 (16)	0.1016 (2)	0.0443 (6)	
N2	0.46313 (10)	0.06819 (19)	0.1157 (3)	0.0676 (8)	
H2A	0.4920	0.0571	0.1503	0.081*	
H2B	0.4534	0.0542	0.0396	0.081*	
N3	0.20298 (9)	0.50200 (15)	0.3904 (2)	0.0426 (6)	
N4	0.04618 (10)	0.60685 (19)	0.4118 (3)	0.0677 (8)	
H4A	0.0161	0.6123	0.3820	0.081*	
H4B	0.0567	0.6223	0.4867	0.081*	
C1	0.19966 (11)	0.3779 (2)	0.0476 (3)	0.0458 (7)	
C2	0.16007 (13)	0.38795 (19)	−0.0621 (3)	0.0527 (8)	
C3	0.11368 (14)	0.3644 (2)	−0.0520 (4)	0.0672 (10)	
C4	0.07795 (15)	0.3761 (2)	−0.1561 (4)	0.0807 (12)	
H4	0.0465	0.3600	−0.1515	0.097*	
C5	0.08796 (16)	0.4107 (3)	−0.2648 (3)	0.0781 (12)	
C6	0.13336 (17)	0.4347 (2)	−0.2702 (3)	0.0790 (13)	
H6	0.1399	0.4598	−0.3424	0.095*	
C7	0.17125 (14)	0.4234 (2)	−0.1717 (3)	0.0624 (9)	
C8	0.10128 (15)	0.3257 (3)	0.0654 (4)	0.1026 (17)	
H8A	0.0669	0.3274	0.0652	0.154*	
H8B	0.1172	0.3525	0.1381	0.154*	
H8C	0.1119	0.2729	0.0675	0.154*	
C9	0.04713 (19)	0.4217 (3)	−0.3750 (4)	0.121 (2)	
H9A	0.0186	0.3952	−0.3574	0.181*	
H9B	0.0570	0.4010	−0.4502	0.181*	
H9C	0.0402	0.4759	−0.3863	0.181*	
C10	0.22098 (18)	0.4523 (4)	−0.1810 (4)	0.0955 (15)	
H10A	0.2278	0.4454	−0.2652	0.143*	
H10B	0.2441	0.4240	−0.1239	0.143*	
H10C	0.2230	0.5063	−0.1597	0.143*	
C11	0.31862 (10)	0.33173 (18)	0.4411 (3)	0.0429 (7)	
C12	0.35379 (10)	0.3143 (2)	0.5561 (2)	0.0437 (7)	
C13	0.40357 (10)	0.3205 (2)	0.5505 (3)	0.0553 (8)	
C14	0.43536 (11)	0.3010 (3)	0.6573 (3)	0.0687 (10)	
H14	0.4684	0.3058	0.6553	0.082*	
C15	0.42023 (13)	0.2751 (2)	0.7651 (3)	0.0631 (10)	
C16	0.37164 (14)	0.2692 (2)	0.7671 (3)	0.0649 (10)	
H16	0.3609	0.2514	0.8399	0.078*	
C17	0.33741 (11)	0.2886 (2)	0.6652 (3)	0.0553 (9)	
C18	0.42314 (14)	0.3460 (3)	0.4338 (4)	0.0879 (14)	
H18A	0.4014	0.3828	0.3890	0.132*	
H18B	0.4262	0.3020	0.3812	0.132*	
H18C	0.4543	0.3696	0.4567	0.132*	
C19	0.45624 (16)	0.2522 (3)	0.8763 (4)	0.0939 (15)	
H19A	0.4399	0.2452	0.9482	0.141*	
H19B	0.4802	0.2920	0.8935	0.141*	
H19C	0.4716	0.2046	0.8583	0.141*	
C20	0.28484 (13)	0.2766 (3)	0.6738 (4)	0.0895 (17)	
H20A	0.2816	0.2466	0.7473	0.134*	
H20B	0.2698	0.2496	0.6005	0.134*	
H20C	0.2694	0.3259	0.6793	0.134*	
C21	0.32081 (11)	0.4560 (2)	0.1842 (3)	0.0495 (8)	
C22	0.36131 (12)	0.5106 (2)	0.1680 (3)	0.0508 (8)	
C23	0.40471 (15)	0.4835 (3)	0.1295 (4)	0.0757 (11)	
C24	0.44281 (16)	0.5350 (3)	0.1284 (5)	0.0941 (15)	
H24	0.4713	0.5166	0.1036	0.113*	
C25	0.44094 (15)	0.6109 (3)	0.1614 (4)	0.0848 (13)	
C26	0.39781 (15)	0.6374 (3)	0.1929 (4)	0.0729 (11)	
H26	0.3949	0.6896	0.2117	0.087*	
C27	0.35870 (12)	0.5894 (2)	0.1974 (3)	0.0606 (9)	
C28	0.41129 (19)	0.4012 (3)	0.0869 (6)	0.117 (2)	
H28A	0.4060	0.3660	0.1526	0.176*	
H28B	0.4436	0.3947	0.0676	0.176*	
H28C	0.3885	0.3906	0.0133	0.176*	
C29	0.48412 (19)	0.6644 (4)	0.1659 (6)	0.125 (2)	
H29A	0.4937	0.6818	0.2505	0.188*	
H29B	0.4756	0.7082	0.1124	0.188*	
H29C	0.5105	0.6371	0.1375	0.188*	
C30	0.31381 (15)	0.6275 (3)	0.2358 (6)	0.1002 (17)	
H30A	0.2854	0.6049	0.1891	0.150*	
H30B	0.3144	0.6819	0.2185	0.150*	
H30C	0.3134	0.6196	0.3240	0.150*	
C31	0.20049 (11)	0.2598 (2)	0.3071 (3)	0.0449 (8)	
C32	0.16422 (12)	0.2020 (2)	0.3347 (3)	0.0522 (8)	
C33	0.13735 (14)	0.2143 (2)	0.4338 (4)	0.0671 (10)	
C34	0.10455 (18)	0.1582 (3)	0.4569 (4)	0.0922 (14)	
H34	0.0868	0.1662	0.5228	0.111*	
C35	0.0967 (2)	0.0922 (3)	0.3890 (6)	0.1039 (17)	
C36	0.12263 (19)	0.0821 (3)	0.2899 (5)	0.0980 (16)	
H36	0.1173	0.0377	0.2410	0.118*	
C37	0.15580 (15)	0.1348 (2)	0.2611 (4)	0.0687 (10)	
C38	0.14293 (17)	0.2838 (3)	0.5166 (4)	0.0919 (14)	
H38A	0.1767	0.2925	0.5453	0.138*	
H38B	0.1262	0.2756	0.5875	0.138*	
H38C	0.1295	0.3281	0.4702	0.138*	
C39	0.0593 (3)	0.0313 (4)	0.4179 (8)	0.193 (4)	
H39A	0.0600	−0.0122	0.3627	0.290*	
H39B	0.0275	0.0538	0.4055	0.290*	
H39C	0.0670	0.0145	0.5034	0.290*	
C40	0.18033 (17)	0.1189 (3)	0.1461 (4)	0.0982 (15)	
H40A	0.1788	0.1644	0.0946	0.147*	
H40B	0.1641	0.0771	0.0988	0.147*	
H40C	0.2135	0.1051	0.1723	0.147*	
C41	0.36040 (10)	0.18707 (19)	0.1549 (3)	0.0455 (7)	
H41	0.3755	0.2140	0.2246	0.055*	
C42	0.38439 (10)	0.12515 (18)	0.1110 (3)	0.0427 (7)	
C43	0.36146 (12)	0.0844 (2)	0.0096 (3)	0.0565 (8)	
H43	0.3766	0.0423	−0.0218	0.068*	
C44	0.31598 (13)	0.1064 (2)	−0.0448 (3)	0.0620 (9)	
H44	0.2998	0.0792	−0.1126	0.074*	
C45	0.29488 (11)	0.1698 (2)	0.0032 (3)	0.0515 (8)	
H45	0.2643	0.1852	−0.0347	0.062*	
C46	0.43379 (11)	0.10668 (19)	0.1795 (3)	0.0501 (8)	
C47	0.15598 (10)	0.51250 (18)	0.3511 (3)	0.0418 (7)	
H47	0.1412	0.4809	0.2868	0.050*	
C48	0.12797 (10)	0.56738 (19)	0.3998 (3)	0.0446 (7)	
C49	0.15054 (12)	0.61396 (19)	0.4959 (3)	0.0533 (8)	
H49	0.1332	0.6520	0.5313	0.064*	
C50	0.19866 (13)	0.6030 (2)	0.5376 (3)	0.0578 (9)	
H50	0.2144	0.6331	0.6025	0.069*	
C51	0.22351 (11)	0.5469 (2)	0.4825 (3)	0.0504 (8)	
H51	0.2563	0.5402	0.5113	0.060*	
C52	0.07610 (11)	0.5748 (2)	0.3426 (3)	0.0523 (8)	

### Atomic displacement parameters (Å^2^)

**Table:**

	*U*^11^	*U*^22^	*U*^33^	*U*^12^	*U*^13^	*U*^23^
Cu1	0.03574 (17)	0.0471 (2)	0.03418 (17)	0.01540 (16)	−0.00220 (12)	−0.00320 (16)
Cu2	0.03820 (18)	0.0504 (2)	0.03404 (16)	0.01698 (16)	−0.00414 (12)	−0.00630 (16)
O1	0.0585 (12)	0.0539 (15)	0.0354 (10)	0.0202 (11)	−0.0096 (9)	−0.0022 (9)
O2	0.0514 (12)	0.0569 (15)	0.0402 (11)	0.0185 (11)	−0.0137 (8)	−0.0064 (10)
O3	0.0450 (11)	0.0702 (15)	0.0398 (10)	0.0234 (12)	−0.0125 (8)	−0.0096 (11)
O4	0.0459 (12)	0.0701 (15)	0.0379 (11)	0.0196 (11)	−0.0085 (9)	−0.0090 (10)
O5	0.0594 (14)	0.0630 (16)	0.0648 (15)	0.0031 (12)	0.0180 (12)	−0.0096 (12)
O6	0.0591 (14)	0.0650 (17)	0.0556 (14)	−0.0006 (11)	0.0117 (11)	−0.0136 (12)
O7	0.0409 (11)	0.0566 (15)	0.0546 (13)	0.0093 (10)	0.0073 (10)	0.0002 (10)
O8	0.0474 (13)	0.0546 (14)	0.0610 (14)	0.0163 (11)	0.0100 (11)	0.0038 (11)
O9	0.0413 (12)	0.0855 (18)	0.0675 (16)	0.0228 (12)	0.0033 (10)	−0.0023 (14)
O10	0.0397 (12)	0.094 (2)	0.0800 (18)	0.0205 (13)	0.0049 (12)	−0.0113 (15)
N1	0.0402 (14)	0.0470 (15)	0.0444 (14)	0.0123 (12)	0.0014 (11)	−0.0111 (12)
N2	0.0439 (15)	0.075 (2)	0.086 (2)	0.0193 (15)	0.0162 (14)	−0.0060 (17)
N3	0.0347 (13)	0.0517 (16)	0.0399 (13)	0.0110 (11)	0.0000 (10)	−0.0049 (11)
N4	0.0443 (15)	0.083 (2)	0.079 (2)	0.0186 (15)	0.0207 (14)	−0.0020 (17)
C1	0.0449 (17)	0.0522 (19)	0.0370 (15)	0.0074 (15)	−0.0050 (12)	0.0058 (14)
C2	0.063 (2)	0.0490 (19)	0.0398 (16)	0.0163 (15)	−0.0152 (14)	−0.0035 (13)
C3	0.063 (2)	0.066 (2)	0.063 (2)	0.0056 (19)	−0.0241 (17)	0.0057 (18)
C4	0.070 (2)	0.070 (3)	0.087 (3)	0.008 (2)	−0.040 (2)	−0.003 (2)
C5	0.099 (3)	0.061 (2)	0.058 (2)	0.024 (3)	−0.043 (2)	−0.009 (2)
C6	0.116 (4)	0.068 (3)	0.0424 (19)	0.020 (2)	−0.026 (2)	0.0018 (17)
C7	0.080 (2)	0.061 (2)	0.0410 (16)	0.013 (2)	−0.0097 (15)	−0.0024 (16)
C8	0.066 (2)	0.138 (5)	0.095 (3)	−0.022 (3)	−0.017 (2)	0.039 (3)
C9	0.132 (4)	0.113 (4)	0.091 (3)	0.038 (4)	−0.077 (3)	−0.015 (3)
C10	0.118 (4)	0.114 (4)	0.055 (2)	0.002 (3)	0.012 (2)	0.013 (2)
C11	0.0367 (15)	0.0481 (18)	0.0405 (15)	0.0096 (13)	−0.0058 (11)	−0.0015 (13)
C12	0.0389 (14)	0.0513 (19)	0.0367 (13)	0.0109 (14)	−0.0093 (11)	0.0003 (14)
C13	0.0410 (16)	0.068 (2)	0.0523 (17)	0.0044 (15)	−0.0076 (13)	−0.0041 (16)
C14	0.0374 (16)	0.090 (3)	0.072 (2)	0.0075 (19)	−0.0146 (14)	−0.018 (2)
C15	0.062 (2)	0.070 (2)	0.0477 (19)	0.0130 (18)	−0.0232 (16)	−0.0085 (16)
C16	0.071 (2)	0.079 (3)	0.0398 (17)	0.010 (2)	−0.0087 (15)	0.0056 (17)
C17	0.0466 (16)	0.074 (3)	0.0427 (16)	0.0091 (16)	−0.0047 (12)	0.0017 (16)
C18	0.056 (2)	0.134 (4)	0.072 (3)	−0.020 (3)	0.0045 (19)	0.002 (3)
C19	0.087 (3)	0.111 (4)	0.069 (3)	0.028 (3)	−0.040 (2)	−0.009 (2)
C20	0.054 (2)	0.150 (5)	0.065 (2)	0.006 (2)	0.0123 (19)	0.023 (3)
C21	0.0439 (17)	0.061 (2)	0.0411 (16)	0.0079 (16)	−0.0030 (13)	0.0019 (16)
C22	0.0495 (17)	0.059 (2)	0.0427 (16)	0.0067 (16)	0.0032 (14)	0.0028 (15)
C23	0.076 (3)	0.081 (3)	0.076 (3)	0.000 (2)	0.030 (2)	−0.005 (2)
C24	0.073 (3)	0.097 (4)	0.123 (4)	−0.001 (3)	0.050 (3)	0.008 (3)
C25	0.065 (2)	0.099 (4)	0.091 (3)	−0.014 (2)	0.015 (2)	0.013 (3)
C26	0.075 (3)	0.072 (3)	0.070 (2)	−0.005 (2)	0.002 (2)	0.001 (2)
C27	0.0500 (19)	0.071 (3)	0.055 (2)	0.0028 (18)	−0.0093 (15)	−0.0002 (18)
C28	0.109 (4)	0.096 (4)	0.165 (5)	0.016 (3)	0.085 (4)	−0.014 (4)
C29	0.095 (4)	0.124 (5)	0.163 (6)	−0.040 (3)	0.040 (4)	0.010 (4)
C30	0.059 (2)	0.064 (3)	0.173 (5)	0.008 (2)	−0.001 (3)	−0.016 (3)
C31	0.0452 (18)	0.055 (2)	0.0322 (14)	0.0157 (15)	−0.0033 (12)	0.0054 (13)
C32	0.0571 (19)	0.052 (2)	0.0453 (17)	0.0112 (16)	0.0001 (14)	0.0084 (15)
C33	0.069 (2)	0.076 (3)	0.059 (2)	0.003 (2)	0.0191 (18)	0.0084 (19)
C34	0.102 (3)	0.104 (4)	0.079 (3)	−0.004 (3)	0.040 (3)	0.015 (3)
C35	0.119 (4)	0.089 (4)	0.110 (4)	−0.030 (3)	0.039 (3)	0.011 (3)
C36	0.116 (4)	0.069 (3)	0.110 (4)	−0.019 (3)	0.019 (3)	−0.003 (3)
C37	0.071 (2)	0.064 (2)	0.070 (2)	0.003 (2)	0.0060 (19)	0.001 (2)
C38	0.102 (3)	0.105 (4)	0.080 (3)	−0.003 (3)	0.052 (3)	−0.012 (3)
C39	0.249 (10)	0.116 (6)	0.240 (10)	−0.080 (6)	0.123 (9)	−0.005 (6)
C40	0.102 (3)	0.103 (4)	0.091 (3)	−0.011 (3)	0.017 (3)	−0.051 (3)
C41	0.0357 (15)	0.0547 (19)	0.0455 (16)	0.0095 (14)	0.0040 (12)	−0.0031 (14)
C42	0.0355 (14)	0.0430 (16)	0.0519 (17)	0.0049 (13)	0.0140 (13)	0.0034 (14)
C43	0.059 (2)	0.0456 (19)	0.067 (2)	0.0119 (16)	0.0185 (17)	−0.0113 (16)
C44	0.058 (2)	0.067 (2)	0.059 (2)	0.0052 (18)	0.0034 (16)	−0.0252 (18)
C45	0.0437 (16)	0.064 (2)	0.0456 (16)	0.0121 (16)	0.0026 (13)	−0.0119 (15)
C46	0.0408 (16)	0.0447 (18)	0.067 (2)	0.0086 (14)	0.0162 (15)	0.0073 (16)
C47	0.0399 (15)	0.0433 (17)	0.0415 (15)	0.0073 (13)	0.0031 (12)	−0.0060 (13)
C48	0.0370 (15)	0.0455 (17)	0.0534 (18)	0.0042 (14)	0.0136 (13)	0.0031 (14)
C49	0.0557 (19)	0.0428 (18)	0.065 (2)	0.0053 (15)	0.0224 (16)	−0.0139 (16)
C50	0.062 (2)	0.057 (2)	0.0554 (19)	−0.0052 (17)	0.0093 (16)	−0.0178 (16)
C51	0.0388 (16)	0.062 (2)	0.0492 (17)	0.0019 (15)	0.0005 (13)	−0.0077 (15)
C52	0.0390 (16)	0.0495 (19)	0.070 (2)	0.0109 (14)	0.0142 (15)	0.0038 (17)

### Geometric parameters (Å, º)

**Table:**

Cu1—Cu2	2.5990 (4)	C19—H19C	0.9600
Cu1—O1	1.9874 (18)	C20—H20A	0.9600
Cu1—O4	1.970 (2)	C20—H20B	0.9600
Cu1—O5	1.958 (2)	C20—H20C	0.9600
Cu1—O7	1.967 (2)	C21—C22	1.502 (5)
Cu1—N3	2.164 (2)	C22—C23	1.415 (5)
Cu2—O2	1.9611 (18)	C22—C27	1.403 (5)
Cu2—O3	1.9671 (18)	C24—C25	1.362 (7)
Cu2—O6	1.983 (3)	C24—C23	1.388 (6)
Cu2—O8	1.981 (2)	C24—H24	0.9300
Cu2—N1	2.165 (2)	C25—C29	1.514 (6)
O1—C1	1.258 (4)	C26—C25	1.375 (6)
O2—C1	1.253 (4)	C26—C27	1.377 (6)
O3—C11	1.254 (3)	C26—H26	0.9300
O4—C11	1.258 (3)	C27—C30	1.523 (6)
O5—C21	1.262 (4)	C28—C23	1.515 (6)
O6—C21	1.248 (4)	C28—H28A	0.9600
O7—C31	1.258 (4)	C28—H28B	0.9600
O8—C31	1.268 (4)	C28—H28C	0.9600
O9—C46	1.222 (4)	C29—H29A	0.9600
O10—C52	1.236 (4)	C29—H29B	0.9600
N1—C41	1.342 (4)	C29—H29C	0.9600
N1—C45	1.336 (4)	C30—H30A	0.9600
N2—H2A	0.8600	C30—H30B	0.9600
N2—H2B	0.8600	C30—H30C	0.9600
N3—C47	1.333 (4)	C31—C32	1.482 (5)
N3—C51	1.323 (4)	C32—C33	1.404 (5)
N4—H4A	0.8600	C32—C37	1.406 (5)
N4—H4B	0.8600	C34—C33	1.380 (6)
C1—C2	1.508 (4)	C34—C35	1.356 (7)
C2—C3	1.377 (5)	C34—H34	0.9300
C2—C7	1.402 (5)	C35—C36	1.382 (7)
C3—C4	1.404 (5)	C35—C39	1.547 (7)
C3—C8	1.512 (6)	C36—H36	0.9300
C4—H4	0.9300	C37—C36	1.366 (6)
C5—C4	1.377 (6)	C37—C40	1.522 (5)
C5—C6	1.343 (6)	C38—C33	1.491 (6)
C5—C9	1.535 (5)	C38—H38A	0.9600
C6—H6	0.9300	C38—H38B	0.9600
C7—C6	1.400 (5)	C38—H38C	0.9600
C7—C10	1.493 (6)	C39—H39A	0.9600
C8—H8A	0.9600	C39—H39B	0.9600
C8—H8B	0.9600	C39—H39C	0.9600
C8—H8C	0.9600	C40—H40A	0.9600
C9—H9A	0.9600	C40—H40B	0.9600
C9—H9B	0.9600	C40—H40C	0.9600
C9—H9C	0.9600	C41—C42	1.381 (4)
C10—H10A	0.9600	C41—H41	0.9300
C10—H10B	0.9600	C42—C43	1.377 (5)
C10—H10C	0.9600	C42—C46	1.502 (4)
C12—C11	1.496 (4)	C43—C44	1.374 (5)
C12—C13	1.404 (4)	C43—H43	0.9300
C12—C17	1.392 (4)	C44—H44	0.9300
C13—C14	1.390 (4)	C45—C44	1.379 (5)
C13—C18	1.505 (5)	C45—H45	0.9300
C14—H14	0.9300	C46—N2	1.321 (4)
C15—C14	1.364 (5)	C47—H47	0.9300
C15—C16	1.364 (5)	C48—C52	1.496 (4)
C15—C19	1.502 (5)	C48—C47	1.379 (4)
C16—H16	0.9300	C48—C49	1.389 (5)
C17—C16	1.388 (4)	C49—C50	1.368 (5)
C17—C20	1.498 (5)	C49—H49	0.9300
C18—H18A	0.9600	C50—C51	1.374 (5)
C18—H18B	0.9600	C50—H50	0.9300
C18—H18C	0.9600	C51—H51	0.9300
C19—H19A	0.9600	C52—N4	1.317 (4)
C19—H19B	0.9600		
			
O1—Cu1—Cu2	82.47 (6)	H19B—C19—H19C	109.5
O4—Cu1—Cu2	85.23 (6)	C17—C20—H20A	109.5
O5—Cu1—Cu2	84.10 (7)	C17—C20—H20B	109.5
O7—Cu1—Cu2	84.47 (7)	C17—C20—H20C	109.5
N3—Cu1—Cu2	176.46 (6)	H20A—C20—H20B	109.5
O4—Cu1—O1	167.66 (9)	H20A—C20—H20C	109.5
O5—Cu1—O1	92.42 (10)	H20B—C20—H20C	109.5
O7—Cu1—O1	86.28 (9)	O5—C21—C22	116.5 (3)
O5—Cu1—O4	87.16 (10)	O6—C21—O5	123.8 (3)
O7—Cu1—O4	91.69 (10)	O6—C21—C22	119.6 (3)
O5—Cu1—O7	168.56 (10)	C23—C22—C21	121.1 (3)
O1—Cu1—N3	93.99 (9)	C27—C22—C21	121.3 (3)
O4—Cu1—N3	98.31 (9)	C27—C22—C23	117.5 (3)
O5—Cu1—N3	96.28 (10)	C22—C23—C28	123.0 (4)
O7—Cu1—N3	95.14 (9)	C24—C23—C22	118.7 (4)
O2—Cu2—Cu1	86.03 (6)	C24—C23—C28	118.3 (4)
O3—Cu2—Cu1	83.25 (7)	C23—C24—H24	118.1
O6—Cu2—Cu1	83.53 (7)	C25—C24—C23	123.8 (4)
O8—Cu2—Cu1	83.33 (7)	C25—C24—H24	118.1
N1—Cu2—Cu1	174.66 (7)	C24—C25—C26	116.9 (4)
O2—Cu2—O3	169.03 (9)	C24—C25—C29	122.4 (4)
O2—Cu2—O6	91.44 (10)	C26—C25—C29	120.7 (5)
O3—Cu2—O6	89.65 (11)	C25—C26—C27	122.3 (4)
O8—Cu2—O6	166.79 (10)	C25—C26—H26	118.8
O2—Cu2—O8	88.92 (9)	C27—C26—H26	118.8
O3—Cu2—O8	87.55 (10)	C26—C27—C22	120.7 (4)
O2—Cu2—N1	98.31 (9)	C26—C27—C30	115.9 (4)
O3—Cu2—N1	92.28 (9)	C22—C27—C30	123.4 (4)
O6—Cu2—N1	99.40 (9)	C23—C28—H28A	109.5
O8—Cu2—N1	93.61 (10)	C23—C28—H28B	109.5
C1—O1—Cu1	122.58 (19)	C23—C28—H28C	109.5
C1—O2—Cu2	120.58 (19)	H28A—C28—H28B	109.5
C11—O3—Cu2	123.63 (18)	H28A—C28—H28C	109.5
C11—O4—Cu1	121.18 (18)	H28B—C28—H28C	109.5
C21—O5—Cu1	123.2 (2)	C25—C29—H29A	109.5
C21—O6—Cu2	121.9 (2)	C25—C29—H29B	109.5
C31—O7—Cu1	122.6 (2)	C25—C29—H29C	109.5
C31—O8—Cu2	122.2 (2)	H29A—C29—H29B	109.5
C41—N1—Cu2	119.8 (2)	H29A—C29—H29C	109.5
C45—N1—Cu2	122.4 (2)	H29B—C29—H29C	109.5
C45—N1—C41	117.6 (3)	C27—C30—H30A	109.5
C46—N2—H2A	120.0	C27—C30—H30B	109.5
C46—N2—H2B	120.0	C27—C30—H30C	109.5
H2A—N2—H2B	120.0	H30A—C30—H30B	109.5
C47—N3—Cu1	117.9 (2)	H30A—C30—H30C	109.5
C51—N3—Cu1	124.7 (2)	H30B—C30—H30C	109.5
C51—N3—C47	117.4 (3)	O7—C31—O8	123.6 (3)
C52—N4—H4A	120.0	O7—C31—C32	118.8 (3)
C52—N4—H4B	120.0	O8—C31—C32	117.5 (3)
H4A—N4—H4B	120.0	C33—C32—C31	120.3 (3)
O1—C1—C2	116.5 (3)	C33—C32—C37	119.2 (3)
O2—C1—O1	125.7 (3)	C37—C32—C31	120.5 (3)
O2—C1—C2	117.8 (3)	C32—C33—C38	123.6 (4)
C3—C2—C1	119.8 (3)	C34—C33—C32	118.1 (4)
C3—C2—C7	121.3 (3)	C34—C33—C38	118.3 (4)
C7—C2—C1	118.8 (3)	C33—C34—H34	118.2
C2—C3—C4	117.6 (4)	C35—C34—C33	123.6 (4)
C2—C3—C8	121.8 (3)	C35—C34—H34	118.2
C4—C3—C8	120.6 (4)	C34—C35—C36	117.4 (4)
C3—C4—H4	118.9	C34—C35—C39	121.7 (5)
C5—C4—C3	122.2 (4)	C36—C35—C39	120.8 (6)
C5—C4—H4	118.9	C35—C36—H36	118.8
C4—C5—C9	119.4 (5)	C37—C36—C35	122.5 (5)
C6—C5—C4	118.6 (3)	C37—C36—H36	118.8
C6—C5—C9	122.0 (4)	C32—C37—C40	123.3 (4)
C5—C6—C7	122.7 (4)	C36—C37—C32	119.2 (4)
C5—C6—H6	118.7	C36—C37—C40	117.5 (4)
C7—C6—H6	118.7	C33—C38—H38A	109.5
C2—C7—C10	121.6 (3)	C33—C38—H38B	109.5
C6—C7—C2	117.6 (4)	C33—C38—H38C	109.5
C6—C7—C10	120.8 (4)	H38A—C38—H38B	109.5
C3—C8—H8A	109.5	H38A—C38—H38C	109.5
C3—C8—H8B	109.5	H38B—C38—H38C	109.5
C3—C8—H8C	109.5	C35—C39—H39A	109.5
H8A—C8—H8B	109.5	C35—C39—H39B	109.5
H8A—C8—H8C	109.5	C35—C39—H39C	109.5
H8B—C8—H8C	109.5	H39A—C39—H39B	109.5
C5—C9—H9A	109.5	H39A—C39—H39C	109.5
C5—C9—H9B	109.5	H39B—C39—H39C	109.5
C5—C9—H9C	109.5	C37—C40—H40A	109.5
H9A—C9—H9B	109.5	C37—C40—H40B	109.5
H9A—C9—H9C	109.5	C37—C40—H40C	109.5
H9B—C9—H9C	109.5	H40A—C40—H40B	109.5
C7—C10—H10A	109.5	H40A—C40—H40C	109.5
C7—C10—H10B	109.5	H40B—C40—H40C	109.5
C7—C10—H10C	109.5	N1—C41—C42	123.0 (3)
H10A—C10—H10B	109.5	N1—C41—H41	118.5
H10A—C10—H10C	109.5	C42—C41—H41	118.5
H10B—C10—H10C	109.5	C41—C42—C46	116.9 (3)
O3—C11—O4	124.9 (3)	C43—C42—C41	118.3 (3)
O3—C11—C12	116.8 (2)	C43—C42—C46	124.8 (3)
O4—C11—C12	118.3 (3)	C42—C43—H43	120.2
C13—C12—C11	119.1 (3)	C44—C43—C42	119.5 (3)
C17—C12—C11	120.4 (2)	C44—C43—H43	120.2
C17—C12—C13	120.4 (3)	C43—C44—C45	118.6 (3)
C12—C13—C18	122.5 (3)	C43—C44—H44	120.7
C14—C13—C12	117.8 (3)	C45—C44—H44	120.7
C14—C13—C18	119.7 (3)	N1—C45—C44	123.0 (3)
C13—C14—H14	118.5	N1—C45—H45	118.5
C15—C14—C13	122.9 (3)	C44—C45—H45	118.5
C15—C14—H14	118.5	O9—C46—N2	123.0 (3)
C14—C15—C19	120.6 (4)	O9—C46—C42	120.4 (3)
C16—C15—C14	117.9 (3)	N2—C46—C42	116.5 (3)
C16—C15—C19	121.5 (4)	N3—C47—C48	124.0 (3)
C15—C16—C17	122.9 (3)	N3—C47—H47	118.0
C15—C16—H16	118.5	C48—C47—H47	118.0
C17—C16—H16	118.5	C47—C48—C49	117.4 (3)
C12—C17—C20	122.6 (3)	C47—C48—C52	118.1 (3)
C16—C17—C12	118.1 (3)	C49—C48—C52	124.5 (3)
C16—C17—C20	119.2 (3)	C48—C49—H49	120.5
C13—C18—H18A	109.5	C50—C49—C48	118.9 (3)
C13—C18—H18B	109.5	C50—C49—H49	120.5
C13—C18—H18C	109.5	C49—C50—C51	119.3 (3)
H18A—C18—H18B	109.5	C49—C50—H50	120.4
H18A—C18—H18C	109.5	C51—C50—H50	120.4
H18B—C18—H18C	109.5	N3—C51—C50	123.0 (3)
C15—C19—H19A	109.5	N3—C51—H51	118.5
C15—C19—H19B	109.5	C50—C51—H51	118.5
C15—C19—H19C	109.5	O10—C52—N4	122.6 (3)
H19A—C19—H19B	109.5	O10—C52—C48	120.1 (3)
H19A—C19—H19C	109.5	N4—C52—C48	117.3 (3)
			
O1—Cu1—Cu2—O2	−10.92 (9)	C1—C2—C3—C4	179.0 (3)
O1—Cu1—Cu2—O3	171.40 (11)	C1—C2—C3—C8	−2.2 (6)
O1—Cu1—Cu2—O6	80.98 (10)	C7—C2—C3—C4	0.9 (6)
O1—Cu1—Cu2—O8	−100.30 (10)	C7—C2—C3—C8	179.7 (4)
O4—Cu1—Cu2—O2	168.21 (10)	C1—C2—C7—C6	−177.6 (3)
O4—Cu1—Cu2—O3	−9.47 (11)	C1—C2—C7—C10	−1.0 (5)
O4—Cu1—Cu2—O6	−99.89 (10)	C3—C2—C7—C6	0.6 (5)
O4—Cu1—Cu2—O8	78.84 (10)	C3—C2—C7—C10	177.1 (4)
O5—Cu1—Cu2—O2	−104.16 (10)	C2—C3—C4—C5	−0.8 (6)
O5—Cu1—Cu2—O3	78.16 (11)	C8—C3—C4—C5	−179.6 (4)
O5—Cu1—Cu2—O6	−12.25 (11)	C6—C5—C4—C3	−0.8 (6)
O5—Cu1—Cu2—O8	166.47 (10)	C9—C5—C4—C3	−180.0 (4)
O7—Cu1—Cu2—O2	76.04 (10)	C4—C5—C6—C7	2.4 (7)
O7—Cu1—Cu2—O3	−101.64 (10)	C9—C5—C6—C7	−178.5 (4)
O7—Cu1—Cu2—O6	167.94 (10)	C2—C7—C6—C5	−2.3 (6)
O7—Cu1—Cu2—O8	−13.33 (9)	C10—C7—C6—C5	−178.9 (4)
Cu2—Cu1—O1—C1	16.1 (2)	C13—C12—C11—O3	49.5 (4)
O4—Cu1—O1—C1	12.1 (6)	C13—C12—C11—O4	−130.2 (3)
O5—Cu1—O1—C1	99.8 (2)	C17—C12—C11—O3	−127.3 (3)
O7—Cu1—O1—C1	−68.8 (2)	C17—C12—C11—O4	53.0 (5)
N3—Cu1—O1—C1	−163.7 (2)	C11—C12—C13—C14	−177.5 (3)
Cu2—Cu1—O4—C11	10.9 (2)	C11—C12—C13—C18	1.6 (6)
O1—Cu1—O4—C11	15.0 (6)	C17—C12—C13—C14	−0.7 (5)
O5—Cu1—O4—C11	−73.4 (2)	C17—C12—C13—C18	178.4 (4)
O7—Cu1—O4—C11	95.2 (2)	C11—C12—C17—C16	176.5 (3)
N3—Cu1—O4—C11	−169.3 (2)	C11—C12—C17—C20	0.4 (6)
Cu2—Cu1—O5—C21	11.1 (3)	C13—C12—C17—C16	−0.2 (5)
O1—Cu1—O5—C21	−71.1 (3)	C13—C12—C17—C20	−176.4 (4)
O4—Cu1—O5—C21	96.6 (3)	C12—C13—C14—C15	1.2 (6)
O7—Cu1—O5—C21	12.1 (7)	C18—C13—C14—C15	−177.9 (4)
N3—Cu1—O5—C21	−165.4 (3)	C16—C15—C14—C13	−0.7 (6)
Cu2—Cu1—O7—C31	13.6 (2)	C19—C15—C14—C13	177.8 (4)
O1—Cu1—O7—C31	96.4 (2)	C14—C15—C16—C17	−0.3 (6)
O5—Cu1—O7—C31	12.6 (7)	C19—C15—C16—C17	−178.9 (4)
O4—Cu1—O7—C31	−71.4 (2)	C12—C17—C16—C15	0.8 (6)
N3—Cu1—O7—C31	−169.9 (2)	C20—C17—C16—C15	177.0 (4)
O1—Cu1—N3—C47	35.0 (2)	O6—C21—O5—Cu1	−0.3 (5)
O1—Cu1—N3—C51	−146.3 (3)	C22—C21—O5—Cu1	−176.7 (2)
O4—Cu1—N3—C47	−144.1 (2)	O5—C21—C22—C23	153.9 (3)
O4—Cu1—N3—C51	34.6 (3)	O5—C21—C22—C27	−22.3 (5)
O5—Cu1—N3—C47	127.9 (2)	O6—C21—C22—C23	−22.6 (5)
O5—Cu1—N3—C51	−53.4 (3)	O6—C21—C22—C27	161.2 (3)
O7—Cu1—N3—C47	−51.6 (2)	C21—C22—C23—C24	−173.5 (4)
O7—Cu1—N3—C51	127.1 (3)	C21—C22—C23—C28	7.8 (6)
Cu1—Cu2—O2—C1	9.9 (2)	C27—C22—C23—C24	2.8 (6)
O3—Cu2—O2—C1	22.1 (7)	C27—C22—C23—C28	−175.9 (4)
O6—Cu2—O2—C1	−73.5 (2)	C21—C22—C27—C26	174.1 (3)
O8—Cu2—O2—C1	93.3 (2)	C21—C22—C27—C30	−5.8 (6)
N1—Cu2—O2—C1	−173.2 (2)	C23—C22—C27—C26	−2.2 (5)
Cu1—Cu2—O3—C11	11.8 (3)	C23—C22—C27—C30	177.8 (4)
O2—Cu2—O3—C11	−0.4 (8)	C25—C24—C23—C22	−0.5 (8)
O6—Cu2—O3—C11	95.4 (3)	C25—C24—C23—C28	178.3 (5)
O8—Cu2—O3—C11	−71.7 (3)	C23—C24—C25—C26	−2.5 (8)
N1—Cu2—O3—C11	−165.3 (3)	C23—C24—C25—C29	176.6 (5)
Cu1—Cu2—O6—C21	18.6 (3)	C27—C26—C25—C24	3.2 (7)
O2—Cu2—O6—C21	104.4 (3)	C27—C26—C25—C29	−175.9 (4)
O3—Cu2—O6—C21	−64.6 (3)	C25—C26—C27—C22	−0.9 (6)
O8—Cu2—O6—C21	13.0 (6)	C25—C26—C27—C30	179.1 (4)
N1—Cu2—O6—C21	−156.9 (3)	O7—C31—C32—C33	−32.4 (4)
Cu1—Cu2—O8—C31	18.5 (2)	O7—C31—C32—C37	146.7 (3)
O2—Cu2—O8—C31	−67.6 (2)	O8—C31—C32—C33	148.2 (3)
O3—Cu2—O8—C31	102.0 (2)	O8—C31—C32—C37	−32.6 (4)
O6—Cu2—O8—C31	24.1 (6)	C31—C32—C33—C34	−179.0 (4)
N1—Cu2—O8—C31	−165.9 (2)	C31—C32—C33—C38	−0.1 (6)
O2—Cu2—N1—C41	168.5 (2)	C37—C32—C33—C34	1.8 (5)
O2—Cu2—N1—C45	−15.9 (3)	C37—C32—C33—C38	−179.3 (4)
O3—Cu2—N1—C41	−14.4 (3)	C31—C32—C37—C36	179.1 (4)
O3—Cu2—N1—C45	161.2 (3)	C31—C32—C37—C40	−3.7 (6)
O6—Cu2—N1—C41	75.6 (3)	C33—C32—C37—C36	−1.8 (6)
O6—Cu2—N1—C45	−108.8 (3)	C33—C32—C37—C40	175.5 (4)
O8—Cu2—N1—C41	−102.1 (3)	C35—C34—C33—C32	−0.3 (8)
O8—Cu2—N1—C45	73.5 (3)	C35—C34—C33—C38	−179.3 (5)
Cu1—O1—C1—O2	−14.3 (4)	C33—C34—C35—C36	−1.3 (9)
Cu1—O1—C1—C2	165.4 (2)	C33—C34—C35—C39	−179.6 (6)
Cu2—O2—C1—O1	−0.7 (4)	C34—C35—C36—C37	1.4 (9)
Cu2—O2—C1—C2	179.6 (2)	C39—C35—C36—C37	179.6 (6)
Cu2—O3—C11—O4	−7.4 (5)	C32—C37—C36—C35	0.1 (8)
Cu2—O3—C11—C12	172.9 (2)	C40—C37—C36—C35	−177.3 (5)
Cu1—O4—C11—O3	−5.7 (4)	N1—C41—C42—C43	−1.5 (5)
Cu1—O4—C11—C12	174.0 (2)	N1—C41—C42—C46	179.3 (3)
Cu2—O6—C21—O5	−16.6 (5)	C41—C42—C43—C44	0.3 (5)
Cu2—O6—C21—C22	159.6 (2)	C46—C42—C43—C44	179.5 (3)
Cu1—O7—C31—O8	−3.9 (4)	C41—C42—C46—O9	22.0 (4)
Cu1—O7—C31—C32	176.79 (19)	C41—C42—C46—N2	−156.7 (3)
Cu2—O8—C31—O7	−14.5 (4)	C43—C42—C46—O9	−157.2 (3)
Cu2—O8—C31—C32	164.9 (2)	C43—C42—C46—N2	24.1 (5)
Cu2—N1—C41—C42	177.1 (2)	C42—C43—C44—C45	0.9 (6)
C45—N1—C41—C42	1.3 (5)	N1—C45—C44—C43	−1.1 (6)
Cu2—N1—C45—C44	−175.6 (3)	C49—C48—C47—N3	−0.2 (5)
C41—N1—C45—C44	0.0 (5)	C52—C48—C47—N3	177.5 (3)
Cu1—N3—C47—C48	179.3 (2)	C47—C48—C49—C50	−0.4 (5)
C51—N3—C47—C48	0.5 (5)	C52—C48—C49—C50	−178.0 (3)
Cu1—N3—C51—C50	−178.9 (3)	C47—C48—C52—O10	−22.2 (5)
C47—N3—C51—C50	−0.2 (5)	C47—C48—C52—N4	158.1 (3)
O1—C1—C2—C3	−79.3 (4)	C49—C48—C52—O10	155.3 (3)
O1—C1—C2—C7	98.9 (4)	C49—C48—C52—N4	−24.4 (5)
O2—C1—C2—C3	100.5 (4)	C48—C49—C50—C51	0.8 (5)
O2—C1—C2—C7	−81.3 (4)	C49—C50—C51—N3	−0.5 (5)

### Hydrogen-bond geometry (Å, º)

**Table:**

*D*—H···*A*	*D*—H	H···*A*	*D*···*A*	*D*—H···*A*
N2—H2*A*···O10^i^	0.86	2.06	2.906 (4)	170
N4—H4*A*···O9^ii^	0.86	2.10	2.955 (4)	173
N4—H4*B*···O9^iii^	0.86	2.44	3.228 (4)	153
C50—H50···O8^iii^	0.93	2.54	3.448 (4)	166

Symmetry codes: (i) *x*+1/2, *y*−1/2, *z*; (ii) *x*−1/2, *y*+1/2, *z*; (iii) −*x*+1/2, *y*+1/2, −*z*+1.
